# Complete remission of advanced MSI-H rectal cancer in a young patient treated with nivolumab: a case report and critical appraisal of the literature

**DOI:** 10.3389/fonc.2025.1680308

**Published:** 2025-11-24

**Authors:** Marian Liberko, Renata Soumarova

**Affiliations:** 1 Department of Oncology, University Hospital Kralovské Vinohrady, Prague, Czechia; 2 Third Faculty of Medicine, Charles University, Prague, Czechia

**Keywords:** rectal cancer, immunotherapy, clinical complete response, non-operative management, case report

## Abstract

Colorectal cancer is a heterogeneous disease, where therapy is chosen based on the presence or absence of predictive and prognostic markers, leading to a higher degree of treatment individualization with the aim of maximizing clinical benefit. In general, microsatellite-unstable tumors are characterized by high sensitivity to immunotherapy. A small percentage of patients with rectal cancer are diagnosed with microsatellite-unstable disease, and immunotherapy therefore represents a potentially curative option. We present a case report of a 34year-old patient with microsatellite-unstable locoregionally advanced rectal cancer who was treated with nivolumab monotherapy. The therapy led to a rapid response, with almost complete clinical remission of the primary tumor reported after only 3 months of therapy. After 6 months of nivolumab therapy, complete clinical remission of the disease was identified, and the patient is currently in a watch-and-wait mode as part of non-surgical management of the disease. The observed toxicity was consistent with the known toxicity profile of immunotherapy and did not lead to discontinuation of therapy. Our case report highlights the need to test for predictive markers in patients with locoregionally advanced rectal cancer in order to identify specific subtypes of the disease that can be treated with immunotherapy with a high probability of achieving clinical complete remission, thereby avoiding potentially risky surgery.

## 1 Introduction

Immunotherapy has completely changed the treatment and prognosis of many malignancies. However, its benefit is not universal and is limited to specific disease subtypes (correlating to a large extent with tumor mutation burden or the presence of neoantigens). In colorectal cancer (CRC), the dMMR/MSI-H phenotype is considered the strongest predictor of benefit from immunotherapy. Tumors with the dMMR/MSI-H phenotype accumulate neoantigens, the new abnormal proteins/peptides that are produced as a consequence of somatic (hypermethylation of the *MLH1* promoter) or germline mutations in mismatch repair proteins (*MLH1, MSH2, MSH6, PMS2*). Neoantigens are then presented either via MHC class I molecules, or can also be presented through MHC II pathways by antigen presenting cells after phagocytosis of tumor cells containing mutated proteins, leading to activation of immunocompetent cells. These tumors are characterized by rich infiltration of CD8+, CD4+ T lymphocytes, macrophages, and NK cells (1, 2).

The frequency of dMMR/MSI-H varies depending on the stage of CRC (3, 4). In stage II, approximately 20% of tumors are dMMR/MSI-H and the presence of dMMR/MSI-H status is associated with a good prognosis, with no indication for adjuvant chemotherapy. In stage III CRC, the prevalence of dMMR/MSI-H decreases and ranges from 10-15%. The prognostic and predictive significance of dMMR/MSI-H in this population is unclear. Approximately 5% of patients with metastatic colorectal cancer (mCRC) are dMMR/MSI-H. This is a small subgroup of patients in whom the presence of dMMR/MSI-H is a negative prognostic marker and these tumors respond poorly to conventional chemotherapy. However, the dMMR/MSI-H phenotype is a positive predictive marker for the efficacy of immunotherapy (5–7).

Approximately 2–3% of rectal tumors are dMMR/MSI-H (7). In the case of dMMR/MSI-H locoregionally advanced rectal cancer, the benefit of standard neoadjuvant chemoradiotherapy is limited, and patients should be treated preferentially with immunotherapy.

Neoadjuvant immune−checkpoint inhibition is emerging as a paradigm−shifting strategy for locally advanced dMMR/MSI−H rectal cancer. Several recent reports have documented remarkably high clinical and pathological response rates in patients with dMMR/MSI-H gastrointestinal malignacies (8–12). Nonetheless, the published experience remains very limited outside of protocolised trial settings, and the majority of real−world cases of MSI−H rectal carcinoma treated exclusively with neoadjuvant monotherapy remain anecdotal. In this manuscript we present a 34−year−old man with low−lying MSI−H rectal cancer treated with neoadjuvant nivolumab monotherapy, who achieved a sustained complete clinical response at 12 months and preserved rectal function without permanent stoma. To our knowledge, this is one of the first documented instances of durable organ−preserving outcome with single−agent nivolumab in this setting, thereby highlighting a potential non‐operative management pathway for selected MSI−H rectal cancers.

## 2 Case presentation

A 34year-old otherwise healthy man was examined since December 2023 for rectal syndrome manifesting mainly as pelvic pressure, flatulence, and urgent bowel movements (up to 10 times a day) and occasional fecal incontinence with blood and mucus was present. Due to these symptoms, the patient lost approximately 10 kg in weight over a period of 6 months. Given the patient’s age and the nature of his symptoms, ulcerative colitis was initially considered as the primary diagnosis. However, treatment for suspected ulcerative colitis did not improve the symptoms and the clinical condition progressed. In January 2024, the patient was referred for a colonoscopy, which revealed a tumor just behind the sphincter, approximately 5–12 cm in size with circular infiltration, mucosal necrosis, and bleeding.

**Figure 1:**
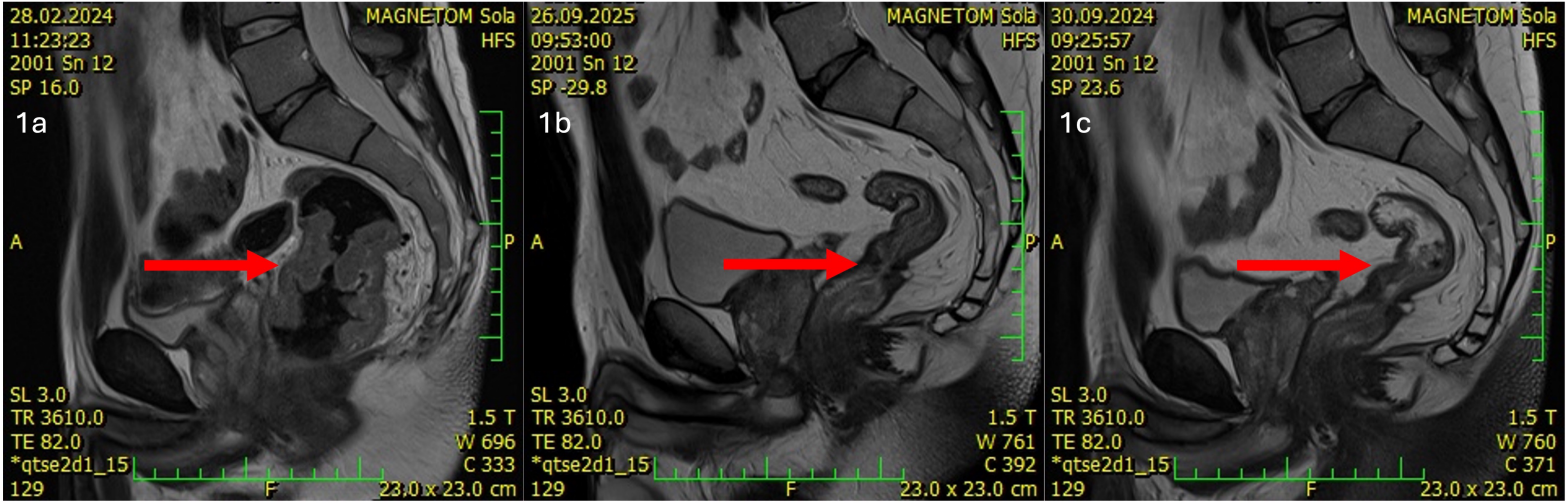
**(a)** Disease status prior to initiation of immunotherapy (magnetic resonance imaging, red arrow showing primary tumor) **(b)** Disease status at the end of immunotherapy (cCR) (magnetic resonance imaging, red arrow showing cCR of primary tumor). **(c)** Disease status 12 months after the end of immunotherapy (persistent cCR) (magnetic resonance imaging, red arrow showing cCR of primary tumor).

During the initial oncology examination in February 2024, the patient was in good overall performance status (PS 0 WHO). Laboratory tests revealed only hypochromic microcytic anemia (hemoglobin 106 g/l, MCV 64 fl, MCH 19.2 pg). Biochemistry was normal, including negative CEA and Ca19–9 markers. Staging was completed, which showed no evidence of distant metastasis. Pelvic MRI confirmed a locally advanced rectal tumor (cT3N2, MRF-, EMVI+): a semicircular tumor predominantly in the middle rectum, extending into the lower rectum and marginally into the upper rectum; the mesorectal fascia was not affected, extramural venous invasion and numerous pelvic lymph nodes were present (**Figure 1a**). Due to the locoregionally advanced disease, the patient was indicated for neoadjuvant therapy.

Before the start of treatment, predictive marker testing was performed, which revealed wtRas, wtBraf, Her2 negat., and MSI-H disease. In parallel, next generation sequencing (NGS) testing of primary tumor biopsy was performed, which revealed a number of alterations (the results are summarized in **Table 1**). Additional genetic testing of the patient did not reveal a germline mutation in the MMR system, leading to sporadic dMMR phenotype.

**Table 1:** Mutations found in NGS testing of primary tumor biopsy.

Gene	Mutation	Type	Pathogenicity	Variant allele frequency (VAF)
PTEN	c.270del	deletion	frameshift	40%
PTEN	c.469dup	duplictation	frameshift	35%
ATM	c.6100C>T	SNV	nonsense	16%
PIK3CA	c.277C>T	SNV	missense	21%
TP53	c.817C>T	SNV	missense	26%
ERBB2	c.2033G>A	SNV	missense	24%
APC	c3385_3386del	deletion	frameshift	40%
APC	c.636del	SNV	synonymous	38%
NOTHC2	c.6125T>C	SNV	missense	15%
CREBBP	c.1447C>T	SNV	nonsense	29%
DICER1	c.4417del	deletion	frameshift	42%
FBXW7	c.1099C>T	SNV	nonsense	27%
KDR	c.1416A>T	SNV	missense	49%
BLM	c.4074G>T	SNV	missense	23%
B2M	c.308del	deletion	frameshift	40%
B2M	c.276del	deletion	frameshift	40%
SDHA	c.17G>C	SNV	missense	35%
PTCH1	c.3606del	deletion	frameshift	37%

MSI-H, 87,8%; TMB, 8,62 mutation/megabase; SNV, single nucleotide variant.

Given the evidence of MSI-H locoregionally advanced rectal cancer, immunotherapy was initiated. The patient started treatment with nivolumab monotherapy at a dose of 240 mg every 14 days in February 2024. The treatment led to a rapid regression of clinical symptoms. Already after two doses of nivolumab, the symptoms of the primary tumor (urgency, tenesmus, meteorism, pain) regressed and hemoglobin levels increased. Re-staging after 3 months of therapy showed significant regression of the primary tumor and lymphadenopathy. According to MRI, near complete remission of the tumor with discrete residue in the rectal wall was described, thus the patient continued with nivolumab. A follow-up MRI scan after 6 months of monotherapy showed clinical complete remission (cCR) of the primary tumor with fibrotic remodeling at the site of the original tumor (**Figure 1b**). Digital rectal examination (DRE) showed no residual disease, with firm, fibrotic tissue dominating at the site of the original tumor. Colonoscopy also confirmed cCR, with a flat scar which was biopsied (histology negative) and lumen stenosis was described with fibrotic remodelling in the area of the original primary rectal tumour (13, 14). With cCR achieved, the patient was indicated for a watch-and-wait approach as part of non-surgical management rectal cancer.

**Figure 2:**
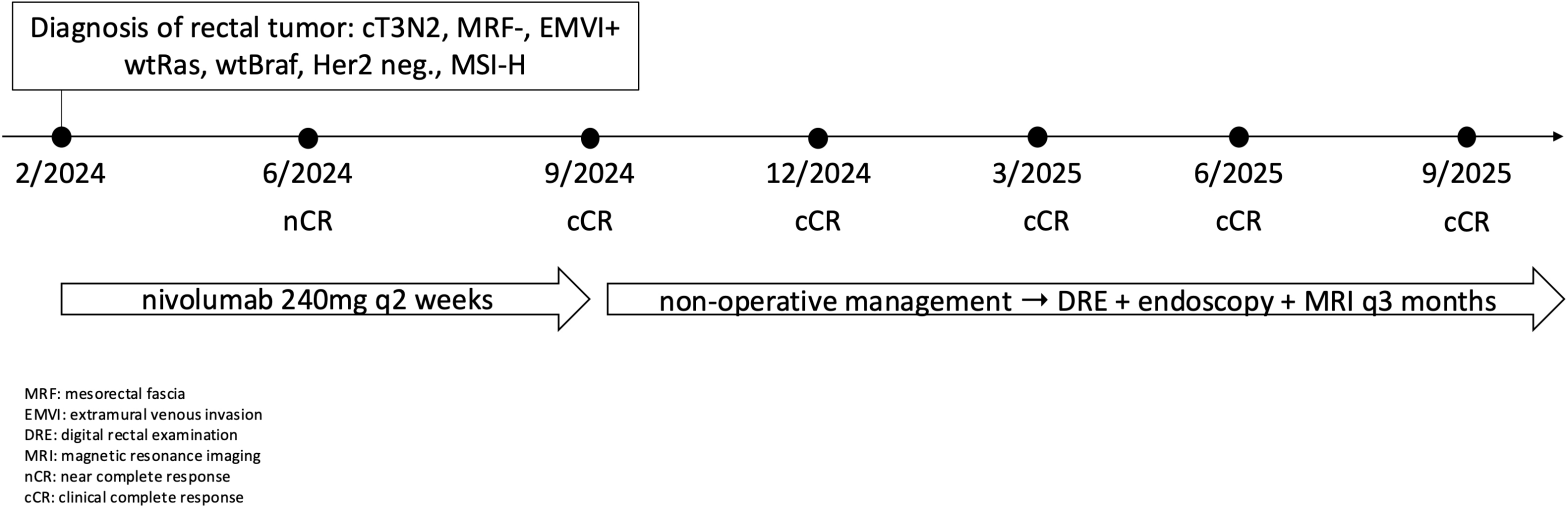
Timeline of patient treatment.

Currently, the patient is 12 months after the last application of nivolumab and is being monitored as part of non-surgical management at 3month intervals using MRI, endoscopy, and DRE. The most recent re-staging took place in September 2025, with cCR persisting (**Figure 1c**). **Figure 2** shows timeline of patient treatment.

### 2.1 Treatment associated toxicity

**Figure 3:**
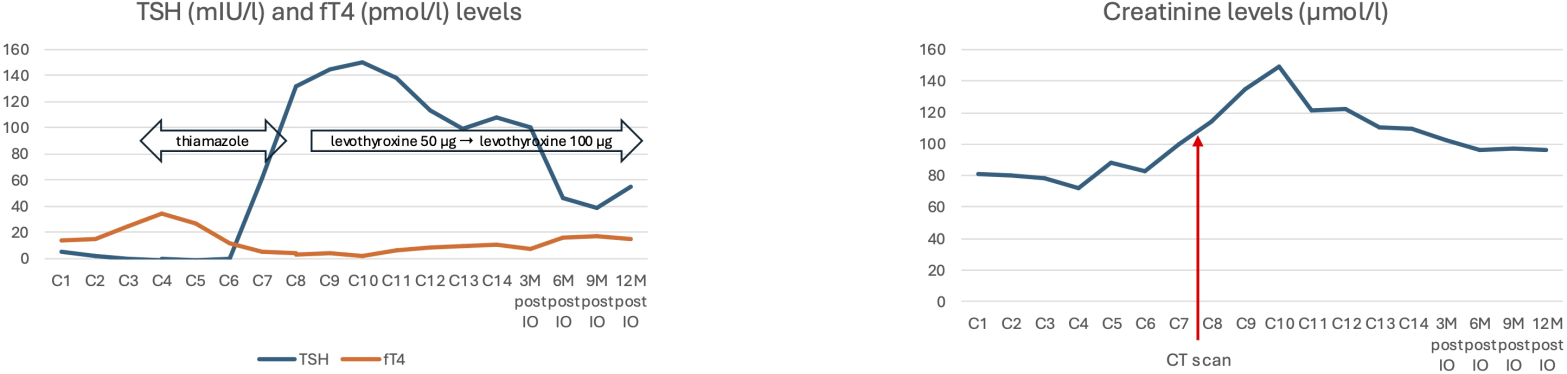
Treatment associated toxicity (endocrine and renal).

Hyperthyroidism G2 was identified during the third cycle of nivolumab, and due to clinical symptoms (sweating, palpitations), therapy with thyreostatics (thiamazole) was initiated, resulting in the regression of clinical symptoms and gradual discontinuation of medication. During the seventh cycle of nivolumab, hypothyroidism G2 was identified, which manifested clinically as increased fatigue. Levothyroxine 50 µg replacement therapy was initiated, followed by escalation to levothyroxine 100 µg, which resulted in normalization of laboratory parameters and resolution of clinical symptoms. In addition to endocrine toxicity, immune-mediated nephritis associated with elevated creatinine (149 µmol/l) was observed. However, the elevation of creatinine occurred following a contrast-enhanced CT scan and therefore a contribution of contrast-induced nephropathy cannot be ruled out. The transient elevation of creatinine was G1, no corticosteroid therapy was initiated, and lifestyle measures led to a spontaneous decrease in creatinine levels to baseline values. **Figure 3** shows the course of endocrine and renal toxicity in the patient.

## 3 Discussion

In this case, the tumor exhibited MSI-H status, as evidenced by instability in 87.8% of microsatellite loci, despite a relatively low tumor mutational burden (8.62 mut/Mb). None of the detected variants involved the exonuclease domains of POLE or POLD1 or the core mismatch-repair genes (MLH1, MSH2, MSH6, PMS2) that are typically responsible for ultramutated phenotypes (15, 16). Truncating alterations in ATM and a missense variant in BLM may reflect impaired DNA-damage-response signaling and some degree of genomic instability but are not expected to markedly increase the number of coding substitutions captured by targeted NGS. The remaining driver mutations (e.g., PTEN, APC, TP53, PIK3CA, ERBB2, FBXW7) are functionally important for tumorigenesis yet have no direct effect on global mutational load (17–19). Furthermore, the bioinformatic pipeline used for tumor mutational burden (TMB) calculation considers only qualifying somatic base substitutions and small indels within the coding footprint of the panel, excluding synonymous, germline, or structural variants. Consequently, multiple detectable alterations can coexist with a numerically low TMB. This profile is therefore consistent with an MSI-H tumor exhibiting a predominant indel signature rather than a true hypermutated genotype. While MSI-H colorectal cancers typically demonstrate markedly elevated mutation rates, variability in TMB values has been reported, influenced by factors such as tumor cellularity, panel size and coverage, and biological heterogeneity of mismatch repair deficiency (20, 21). Importantly, MSI-H status alone is a robust biomarker of defective mismatch repair and has established diagnostic, prognostic, and therapeutic implications, including sensitivity to immune checkpoint inhibition, independent of TMB level (22).

In a study by Cercek et al., 6 of 21 patients (29%) with dMMR/MSI-H rectal cancer treated with neoadjuvant chemotherapy had disease progression, while progression was not reported in any of the 63 patients with proficient mismatch repair/microsatellite stable (pMMR/MSS) cancer. On the other hand, both dMMR/MSI-H and pMMR/MSS carcinomas were sensitive to neoadjuvant chemoradiotherapy (23). On the other hand, data are also available showing lower sensitivity of dMMR/MSI-H tumors to neoadjuvant chemoradiotherapy. In an analysis of 5,086 patients with rectal cancer treated with neoadjuvant chemoradiotherapy, 4,450 pMMR/MSS and 636 dMMR/MSI-H patients were identified. Patients with dMMR/MSI-H rectal cancer had a significantly lower pCR rate compared to pMMR/MSS cancers (5.9% vs. 8.9%, p=0.01) (24). At the same time, dMMR/MSI-H rectal carcinomas treated with neoadjuvant chemoradiotherapy show a lower response to therapy in terms of disease downstaging (25).

Given the unprecedented effect of immunotherapy in dMMR/MSI-H mCRC, there is a trend to move immunotherapy to the treatment of early stages of CRC, when the negative impact of cancer on the immune system is expected to be lower (26). The clear benefit of neoadjuvant immunotherapy in the treatment of dMMR/MSI-H CRC has been demonstrated in the NICHE-1 (pCR 60%) (8), NICHE-2 (pCR 68%) (9), and NICHE-3 (pCR 68%) (10), where high cCR and pCR rates have opened the discussion on possible non-surgical management of these patients. The benefit of neoadjuvant immunotherapy has also been demonstrated in non-colorectal malignancies. Specifically, in the case of dMMR/MSI-H gastric cancer, the GERCOR-NEONIPIGA study reported a pCR rate of 58.6% (11) of patients, and similarly, in the INFINITY study, the pCR rate was 60% (12).

In rectal cancer, oncological treatment (neoadjuvant chemotherapy, chemoradiotherapy, or total neoadjuvant therapy) in combination with surgery is associated with morbidity, rectal syndrome, and a deterioration in patients’ quality of life. The high percentage of cCR and pCR observed with immunotherapy has led to interest in testing immunotherapy in a population of patients with dMMR-MSI-H carcinomas (27). We have data from a phase II monocentric study in which patients with dMMR/MSI rectal cancer were treated with 6 months of monotherapy with the anti-PD-1 drug dostarlimab. cCR was defined as pCR in the case of surgery or absence of tumor according to MRI, endoscopic, and per rectum examination >12 months after the end of therapy (28). Updated data confirm these results, with all 49 patients with dMMR/MSI-H rectal cancer who underwent complete 6month therapy with dostarlimab achieved cCR and continued with non-surgical management. Of these, 37 patients had persistent cCR at 12 months and 2year recurrence-free survival was 96% (29).

The optimal duration of neoadjuvant immunotherapy varies depending on the study and tumor type and is unknown. An analysis evaluating the duration of immunotherapy and achievement of cCR was recently published (30). The probability of achieving cCR increases with the duration of immunotherapy (it occurs more rapidly when anti-PD-1 and anti-CTLA4 are combined), thus increasing the chance of non-operative management of patients (30). However, it is always necessary to optimize the duration of immunotherapy individually in order to maximize clinical benefit while minimizing potential immune-related toxicity.

With regard to the effect of neoadjuvant immunotherapy and the achievement of cCR, data on the duration of response in patients with dMMR/MSI-H locally advanced rectal carcinomas are available from a number of studies. Cercek et al. recently published the results of a cohort of 49 patients treated with dostarlimab. All 49 patients (100%) who completed the full 6-month course of dostarlimab therapy achieved cCR and were enrolled in non-surgical management. Thirty-seven of the 49 patients had ongoing cCR 12 months after the end of therapy. Recurrence-free survival at 2 years was 96%; the median follow-up for recurrence was 30.2 months (29). Encouraging results in terms of clinical response and duration of response were presented in a cohort of 24 patients who were treated with anti-PD1 therapy with curative intent and were followed for at least 12 months after achieving cCR. The median duration of treatment was 6 months. No local regrowth or distant metastasis was observed in a median follow-up time of 29.1 months after cCR. The 3-year disease-free and overall survivals were both 100% (31). Similarly, the effect of neoadjuvant immunotherapy is also documented in a cohort of 17 patients with dMMR/MSI-H locally advanced rectal cancer. Fourteen of the 17 patients completed 6 months of therapy. A total of 16 of the 17 (94.1%) patients achieved cCR and were treated with non-surgical management. At a median follow-up of 9.6 months, no local recurrence was observed (32). These results are confirmed by a recently published systematic review of 12 studies (prospective phase I and II studies), where cCR in the population of dMMR/MSI-H patients with locally advanced rectal cancer ranged from 56 to 100% (33). The most comprehensive analysis includes 19 studies comprising 504 patients. PD-1 monotherapy was associated with high cCR 75–100%, with organ preservation achieved in up to 100% of patients. Long-term follow-up confirmed durable disease control in nonoperatively managed cohorts. Across all treatment strategies, the pooled cCR rate was 82.4%. In the subgroup of patients managed non-operatively after achieving a cCR, the pooled 2–3year DFS was 100% with no local regrowth or distant recurrences reported during follow-up (34).

Despite the relatively small number of patients, the heterogeneity of individual studies, and the short follow-up period, it is clear that neoadjuvant immunotherapy is a potentially curative treatment method in patients with dMMR/MSI-H locally advanced rectal carcinomas and, in a significant percentage of patients, leads to the possibility of a non-surgical management associated with a improved quality of life. Long-term disease control with currently limited follow-up time is also very promising, but longer follow-up results are needed to increase the robustness of the findings. The management of patients with local and/or distant disease relapse after initial cCR (re-challenge with monotherapy? re-challenge with a combination of CTLA4 inhibitors)? remains an unresolved issue at present.

Our case adds meaningful insight into the evolving landscape of neoadjuvant immunotherapy for dMMR/MSI−H rectal cancer. While prior reports have primarily focused on trial populations treated with dostarlimab or combination regimens (PD−1 plus CTLA−4 blockade) in highly selected centres, our patient underwent single−agent nivolumab in a non−trial, clinical‐routine context. Moreover, our report documents a prolonged clinical complete response of at least 12 months with avoidance of abdominoperineal resection and permanent stoma, thereby underscoring the potential for organ preservation in a younger patient with low−lying tumour. Given the profound implications for quality of life (bowel, urinary, sexual function) in this population, the case underscores the importance of MSI testing in rectal cancer and supports further investigation of non−operative management strategies. While the durability beyond 12 months remains to be fully characterised, our experience suggests that neoadjuvant PD−1 monotherapy may be a feasible, tolerable, and function−preserving option in selected MSI−H rectal cancers.

## 4 Conclusion

Given the unprecedented effect of neoadjuvant immunotherapy in patients with dMMR/MSI-H rectal cancer, it is essential to determine MMR protein expression in all patients and, if dMMR/MSI-H status is confirmed, to initiate immunotherapy followed by non-surgical management if cCR is achieved.

## 5 Patient perspective

When I was diagnosed with low-lying rectal cancer at the age of 34, I was told that the standard treatment would most likely require removal of the rectum and a permanent stoma. The idea of living with a stoma at such a young age was very difficult for me to accept. When my doctors explained that, due to the MSI-H status of my tumor, I might be eligible for immunotherapy with the possibility of avoiding surgery altogether, I was very eager to start this treatment.

Soon after the therapy began, I noticed a rapid relief of my symptoms — the bleeding and discomfort disappeared within weeks. I experienced almost no side effects throughout the treatment, which made it easier to continue working and maintain my normal daily life. After three months, imaging already showed a near-complete response, and after six months, I was told that I had achieved a clinical complete response.

Now, more than a year after finishing treatment, I remain in complete clinical remission without any signs of recurrence. I am very grateful for the opportunity to keep my rectum and avoid a permanent stoma. Even though I have to undergo frequent imaging and endoscopic evaluations, I consider it a small price to pay for preserving normal function and quality of life.

## Data Availability

The original contributions presented in the study are included in the article/supplementary material. Further inquiries can be directed to the corresponding author.
